# Development and validation of a clinical breast cancer tool for accurate prediction of recurrence

**DOI:** 10.1038/s41523-024-00651-5

**Published:** 2024-06-15

**Authors:** Asim Dhungana, Augustin Vannier, Fangyuan Zhao, Jincong Q. Freeman, Poornima Saha, Megan Sullivan, Katharine Yao, Elbio M. Flores, Olufunmilayo I. Olopade, Alexander T. Pearson, Dezheng Huo, Frederick M. Howard

**Affiliations:** 1 Pritzker School of Medicine, University of Chicago, Chicago, IL USA; 2 Department of Public Health Sciences, University of Chicago, Chicago, IL USA; 3 Department of Medicine, NorthShore University HealthSystem, Evanston, IL USA; 4 Department of Pathology, NorthShore University HealthSystem, Evanston, IL USA; 5 Department of Surgery, NorthShore University HealthSystem, Evanston, IL USA; 6 Department of Pathology, Ingalls Memorial Hospital, Harvey, IL USA; 7 Department of Medicine, University of Chicago, Chicago, IL USA

**Keywords:** Breast cancer, Prognostic markers, Breast cancer, Prognostic markers

## Abstract

Given high costs of Oncotype DX (ODX) testing, widely used in recurrence risk assessment for early-stage breast cancer, studies have predicted ODX using quantitative clinicopathologic variables. However, such models have incorporated only small cohorts. Using a cohort of patients from the National Cancer Database (NCDB, *n* = 53,346), we trained machine learning models to predict low-risk (0-25) or high-risk (26-100) ODX using quantitative estrogen receptor (ER)/progesterone receptor (PR)/Ki-67 status, quantitative ER/PR status alone, and no quantitative features. Models were externally validated on a diverse cohort of 970 patients (median follow-up 55 months) for accuracy in ODX prediction and recurrence. Comparing the area under the receiver operating characteristic curve (AUROC) in a held-out set from NCDB, models incorporating quantitative ER/PR (AUROC 0.78, 95% CI 0.77–0.80) and ER/PR/Ki-67 (AUROC 0.81, 95% CI 0.80–0.83) outperformed the non-quantitative model (AUROC 0.70, 95% CI 0.68–0.72). These results were preserved in the validation cohort, where the ER/PR/Ki-67 model (AUROC 0.87, 95% CI 0.81–0.93, *p* = 0.009) and the ER/PR model (AUROC 0.86, 95% CI 0.80–0.92, *p* = 0.031) significantly outperformed the non-quantitative model (AUROC 0.80, 95% CI 0.73–0.87). Using a high-sensitivity rule-out threshold, the non-quantitative, quantitative ER/PR and ER/PR/Ki-67 models identified 35%, 30% and 43% of patients as low-risk in the validation cohort. Of these low-risk patients, fewer than 3% had a recurrence at 5 years. These models may help identify patients who can forgo genomic testing and initiate endocrine therapy alone. An online calculator is provided for further study.

## Introduction

Breast cancer is the most common form of cancer in the US, with nearly 300,000 new cases diagnosed in 2022^1^. Hormone receptor positive (HR + ) breast cancer constitutes about 70% of newly diagnosed cases and is generally treated effectively with hormonal therapy, but a subset of more aggressive disease requires treatment with chemotherapy^2^. The Oncotype DX (ODX) test is a 21-gene expression assay that assigns patients a risk recurrence score from 0 to 100 to identify cases of HR + /HER2- breast cancer that would most likely benefit from adjuvant chemotherapy^3^. ODX has been extensively validated^3–6^ and is currently recommended by national guidelines to identify patients with HR + /HER2- breast cancer with up to 3 lymph nodes involved who require chemotherapy^7,8^. Though a valuable clinical tool, ODX costs ~$4000 per test in the US, which may reduce accessibility in low-resource settings—especially internationally where breast cancer accounts for another 2 million new cases each year^9^. In the US, racial disparities exist in ODX testing uptake, and several studies have demonstrated higher risks of recurrence among racial/ethnic minority groups, particularly Black and Hispanic, even in patients with low ODX scores^10–13^. Furthermore, genomic testing is time-consuming and can contribute to delays in administration of adjuvant treatment^14^. Testing is performed in over 50% of newly diagnosed HR + /HER2- breast cancer cases nationwide with this percentage rising, and as such more tumors with low-risk clinical features are undergoing ODX testing.

There have been several attempts to use routinely available clinical features to predict likelihood of a high ODX risk score. A set of equations derived from patient data at Magee-Women’s hospital established linear relationships between quantitative pathologic parameters and the numeric ODX score^15^, and these equations have been validated in various settings^16^. However, formulas that account for the changing practice patterns of ODX testing are needed. The National Cancer Database (NCDB) is a hospital-based registry that includes data from ~70% of new invasive cancer diagnoses in the US and is the ideal dataset for such models^17^. Using patient data captured by the NCDB, machine learning methods have been developed for the prediction of high-risk ODX status based on readily available clinicopathologic features such as patient age, tumor size, grade, HR status, and histologic type^18–20^. For example, a nomogram developed at the University of Tennessee Medical Center using data from the NCDB has been evaluated in various contexts^21–23^. However, until recently, the NCDB did not report quantitative histologic parameters for variables such as estrogen receptor (ER), progesterone receptor (PR), and Ki-67 expression in breast cancer patients, limiting the accuracy of such models. As such, we trained machine learning models in the NCDB incorporating these quantitative histologic variables. We then validated the models in a large and diverse patient cohort from the University of Chicago Medical Center (UCMC), assessing both their predictive accuracy for ODX and correlation with survival outcomes.

## Results

### Cohort description

We performed preliminary assessment of the univariate accuracy of potential features in the NCDB to identify a minimum feature set—HER2 copy number and HER2/CEP17 ratio were excluded as potential features due to low predictive accuracy and limited availability (Supplementary Fig. 1). From the NCDB cohort, we identified 53,346 patients with HR + /HER2- Stage I-III breast cancer with three or fewer lymph nodes involved and no missing data for candidate features. Patients were predominantly non-Hispanic White (80.0%) with a mean age of 60 years, and a median follow-up time of 28 months (Table 1). With this short follow-up, only 1% (*n* = 488) of patients had passed away. All included patients had ODX testing, 7% (*n* = 3815) with high-risk (score ≥ 26) results.

**Table 1:** Baseline demographics from the National Cancer Database Cohort

		Missing	Overall	High Oncotype Dx Scores (26-100)	Low Oncotype Dx Scores (0-25)	*p*-value
***n***			53346	3815	49531	
**Age, mean (SD)**		0	60.2 (10.9)	60.4 (11.3)	60.1 (10.8)	0.185
**Sex,** ***n*** **(%)**	Female	0	52906 (99.2)	3783 (99.2)	49123 (99.2)	0.995
	Male		440 (0.8)	32 (0.8)	408 (0.8)	
**Race/Ethnicity,** ***n*** **(%)**	Asian	0	2466 (4.6)	201 (5.3)	2265 (4.6)	<0.001
	Hispanic		3437 (6.4)	257 (6.7)	3180 (6.4)	
	Native American		191 (0.4)	17 (0.4)	174 (0.4)	
	Non-Hispanic Black		4188 (7.9)	378 (9.9)	3810 (7.7)	
	Non-Hispanic White		42683 (80.0)	2927 (76.7)	39756 (80.3)	
	Other		381 (0.7)	35 (0.9)	346 (0.7)	
**Charlson/Deyo Score,** ***n*** **(%)**	0	0	44525 (83.5)	3121 (81.8)	41404 (83.6)	0.005
	>=1		8821 (16.5)	694 (18.2)	8127 (16.4)	
**Grade,** ***n*** **(%)**	1.0	0	14552 (27.3)	398 (10.4)	14154 (28.6)	<0.001
	2.0		32401 (60.7)	2060 (54.0)	30341 (61.3)	
	3.0		6393 (12.0)	1357 (35.6)	5036 (10.2)	
**Histologic Subtype,** ***n*** **(%)**	Ductal	0	41847 (78.4)	3218 (84.4)	38629 (78.0)	<0.001
	Ductal and Lobular		2484 (4.7)	145 (3.8)	2339 (4.7)	
	Lobular		7828 (14.7)	411 (10.8)	7417 (15.0)	
	Mucinous		952 (1.8)	35 (0.9)	917 (1.9)	
	Others		235 (0.4)	6 (0.2)	229 (0.5)	
**Tumor Size (mm), mean (SD)**		0	18.1 (15.9)	20.2 (21.9)	17.9 (15.3)	<0.001
**Lymph Node Status,** ***n*** **(%)**	Negative	0	43643 (81.8)	3068 (80.4)	40575 (81.9)	0.022
	Positive		9703 (18.2)	747 (19.6)	8956 (18.1)	
**Lymphovascular Invasion,** ***n*** **(%)**	Absent	0	46224 (86.6)	3069 (80.4)	43155 (87.1)	<0.001
	Present		7122 (13.4)	746 (19.6)	6376 (12.9)	
**Stage Group,** ***n*** **(%)**	1.0	0	49778 (93.3)	3224 (84.5)	46554 (94.0)	<0.001
	2.0		3396 (6.4)	563 (14.8)	2833 (5.7)	
	3.0		172 (0.3)	28 (0.7)	144 (0.3)	
**Receptor Status,** ***n*** **(%)**	ER + PR+	0	49610 (93.0)	3043 (79.8)	46567 (94.0)	<0.001
	ER + PR-		3700 (6.9)	771 (20.2)	2929 (5.9)	
	ER-PR+		36 (0.1)	1 (0.0)	35 (0.1)	
**ER (% Positive), mean (SD)**		0	93.7 (10.4)	91.2 (14.5)	93.9 (10.0)	<0.001
**PR (% Positive), mean (SD)**		0	69.5 (34.4)	41.8 (37.6)	71.6 (33.2)	<0.001
**Ki67 (% Positive), mean (SD)**		0	16.7 (14.6)	27.4 (19.9)	15.9 (13.8)	<0.001
**Chemotherapy,** ***n*** **(%)**	Chemotherapy	241	5477 (10.3)	2579 (68.2)	2898 (5.9)	<0.001
	No Chemo		47628 (89.7)	1205 (31.8)	46423 (94.1)	
**Hormonal Therapy,** ***n*** **(%)**	Hormonal Therapy	837	49298 (93.9)	3475 (93.3)	45823 (93.9)	0.159
	No Hormonal Therapy		3211 (6.1)	248 (6.7)	2963 (6.1)	
**Vital Status,** ***n*** **(%)**	Alive	18062	34796 (98.6)	2415 (98.2)	32381 (98.7)	0.061
	Deceased		488 (1.4)	45 (1.8)	443 (1.3)	

The UCMC cohort with similar inclusion criteria had 970 patients and was more diverse, with 30.8% non-Hispanic Black patients, although most were still non-Hispanic White (61.4%). Patients who met our inclusion criteria had a mean age of 58 years and a longer median follow-up time of 55 months. Of these patients, 305 had ODX testing, and 18% (*n* = 56) had high-risk results, and 29 recurrence events were documented (Supplementary Table 1). Patients without ODX testing were used to evaluate long-term outcomes of patients based on model predictions.

### Model development and performance assessment

To allow applicability in settings where certain markers may be unavailable, we developed models that only incorporated routinely available clinical features without quantitative immunohistochemistry, then added quantitative ER/PR status, and finally quantitative ER/PR/Ki-67 status. A subset of 80% of the data from NCDB was used for hypermeter optimization and feature selection. A grid search was performed to select the optimal model and hyperparameters, and logistic regression was chosen as the base model. Sequential forward feature selection identified the most informative features to include in each model (Supplementary Fig. 2). All models incorporated grade, PR status or percent, and ductal histologic subtype. Grade, PR status, and Ki-67% (when available) had the greatest contributions to model performance. Furthermore, models were compared to a previously published deep learning model predicting ODX from digital histology^24^—but this was only performed in the validation cohort as digital histology was not available in the NCDB.

**Fig. 1:**
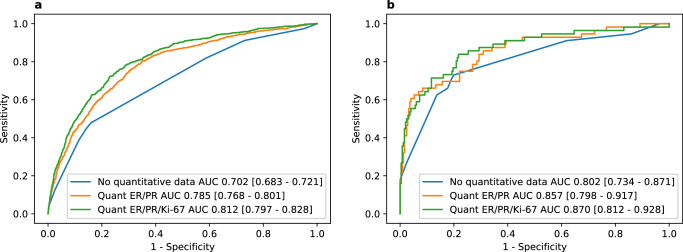
Predictive accuracy for high-risk recurrence score. **a** Receiver operating characteristic curves for prediction of high Oncotype DX using the non-quantitative, quantitative ER/PR, and quantitative ER/PR/Ki-67 models in the National Cancer Database held-out test cohort (*n* = 10,670). **b** The same curves plotted for the external University of Chicago Medical Center validation cohort (*n* = 305).

When comparing the area under the receiver operating characteristic curve (AUROC) in the held-out NCDB test cohort, the quantitative ER/PR model (AUROC 0.78, 95% CI 0.77–0.80) and the ER/PR/Ki-67 model (AUROC 0.81, 95% CI 0.80–0.83) both performed better than the non-quantitative model (AUROC 0.70, 95% CI 0.68–0.72, Fig. 1, Table 2). Quantitative models had greater correlation between model predictions and true ODX score in the held-out test set from NCDB with a Pearson correlation coefficient of 0.43 for the quantitative ER/PR/Ki-67 model, and the slope of calibration curves for quantitative models was >0.90 indicating good calibration (Supplementary Fig. 3). Although we designed models based on the availability (or lack thereof) of quantitative IHC results, we also evaluated performance with missing data for other input values using mean imputation to replace values in the testing cohort to simulate missing data (Supplementary Table 2). Missing data for most input variables resulted in decreased model performance, aside from age, tumor size, and quantitative ER status—but meaningful predictions (with AUROC exceeding random chance) could still be made with any single missing variable. We also evaluated performance in select patient subgroups to ensure consistent results in different populations in the held-out NCDB test set. Across models—performance was better in ductal and mixed ductal/lobular tumors, and worse in lobular or mucinous tumors—other histologic subtypes were not evaluated due to small number of cases (Supplementary Table 3). We found no meaningful difference in racial/ethnic subgroups and node-negative versus node-positive cases (Supplementary Tables 4 and 5). In all cases, regardless of subgroup, the ER/PR/Ki-67 model performed the best. When validating the trained models on the subset of the external UCMC dataset with ODX available (*n* = 305), overall AUROCs were largely preserved (Table 2), with a statistically significant improvement seen in the ER/PR/Ki-67 quantitative model (AUROC 0.87, 95% CI 0.81–0.93, *p* = 0.009) and the ER/PR model (AUROC 0.86, 95% CI 0.80–0.92, *p* = 0.031) over the non-quantitative model (AUROC 0.80, 95% CI 0.73–0.87). In the subset of patients who also had digital pathology available (*n* = 253), AUROC of the performance of the quantitative ER/PR/Ki-67 model (0.86, 95% CI 0.79–0.93) was similar to our previously published deep learning pathologic model (AUROC 0.85, 95% CI 0.78–0.92), suggesting that if quantitative immunohistochemistry is unavailable, this deep learning model may be a reasonable surrogate (Supplementary Fig. 4).

**Table 2:** Model performance characteristics

National Cancer Database, Held-Out Test Cohort				
Model	AUROC (CI)	AUPRC (CI)	z-statistic	*p*-value (compared to ‘No quantitative data’ model)
**No quantitative data**	0.70 (0.68–0.72)	0.21 (0.18–0.23)	–	–
**Quant ER/PR**	0.78 (0.77–0.80)	0.24 (0.21–0.27)	11.12	<0.001
**Quant ER/PR/Ki-67**	0.81 (0.80–0.83)	0.28 (0.25–0.32)	14.54	<0.001

University of Chicago Medical Center, validation cohort				
**No quantitative data**	0.80 (0.73–0.87)	0.60 (0.48–0.72)	–	–
**Quant ER/PR**	0.86 (0.80–0.92)	0.69 (0.58–0.80)	2.16	0.031
**Quant ER/PR/Ki-67**	0.87 (0.81–0.93)	0.71 (0.58–0.82)	2.62	0.009

Results are listed for the area under the receiver operating characteristic curve (AUROC) and area under the precision recall curve (AUPRC) for prediction of high-risk Oncotype DX recurrence score. Confidence intervals and *p*-values were generated using Delong’s method for AUROC, and confidence intervals for AUPRC were generated using 1000 iteration bootstrapping.

To enhance the clinical utility of these models we selected cutoffs which achieved 90% and 95% sensitivity for high ODX in the NCDB training cohort to facilitate the use of these models as rule-out tests—i.e., identifying patients who can forgo ODX (Supplementary Table 6). Inclusion of additional quantitative features consistently increased model specificity at each threshold—with a specificity of 54% for the quantitative ER/PR/Ki-67 model seen in the validation cohort at the target 90% sensitivity threshold. To facilitate the study of the presented models, we developed an online calculator to compute and display model predictions with respect to these proposed thresholds for low / very low risk patients, available at rsncdb.cri.uchicago.edu (Supplementary Fig. 5).

### Survival analysis

**Fig. 2:**
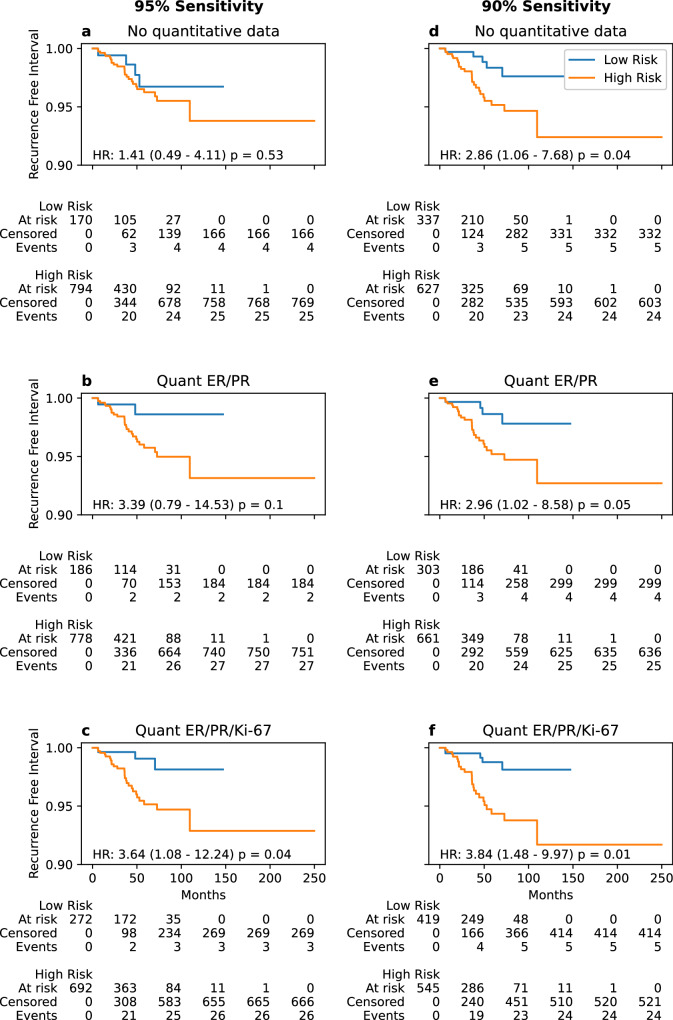
Recurrence Rates Stratified by Model Prediction. Kaplan–Meier curves are shown for the recurrence-free intervals of patients (*n* = 964) in the University of Chicago Medical Center cohort classified as low- and high-risk by the (**a**) non-quantitative model, (**b**) quantitative ER/PR model, and (**c**) quantitative ER/PR/Ki-67 model using a 95% sensitivity cutoff for high-risk disease to stratify patients. Survival analysis results are repeated at the 90% sensitivity cutoff for these same models, shown in (**d**–**f**) respectively.

Follow-up was too short to appreciate meaningful differences in survival based on model predictions in the NCDB cohort (Supplementary Table 7). In the UCMC dataset, the quantitative ER/PR/Ki-67 model predictions had greater concordance with recurrence-free interval (c-index 0.71, adjusted hazard ratio [aHR] 1.43, 95% CI 1.11–1.85, *p* = 0.01) than the quantitative ER/PR model (c-index 0.69, aHR 1.44, 95% CI 1.09–1.89, *p* = 0.01) or the non-quantitative model (c-index 0.66, aHR 1.33, 95% CI 1.03–1.73, *p* = 0.03, Table 3). There was a trend towards association with recurrence-free survival for the ER/PR/Ki-67 model predictions (aHR 1.24, 95 % CI 0.98–1.57, *p* = 0.07). When applying the 90% sensitivity thresholds to the UCMC dataset, patients identified as high risk by all models had a shorter recurrence free interval, with the largest hazard ratio seen for the ER/PR/Ki-67 model (aHR 3.84, 95% CI 1.48–9.97, *p* = 0.01, Fig. 2). At the 95% sensitivity threshold, patients identified as high risk by the ER/PR/Ki-67 model had a significantly shorter recurrence-free interval (aHR 3.64, 95% CI 1.08–12.24, *p* = 0.04), with a trend towards shorter recurrence-free interval in high-risk patients per the quantitative ER/PR model (aHR 3.39, 95% CI 0.79–14.53, *p* = 0.10). Long-term recurrence rates were <3% for quantitative models at both cutoffs, although the ER/PR/Ki-67 model identified more patients as low risk; with 43% (*n* = 419 out of 964) identified as low risk at the 90% sensitivity threshold. Furthermore, in the subset of patients with digital pathology available (*n* = 670), both our previous deep learning model on digital histology and the quantitative models provided complementary information (Supplementary Fig. 6). Patients predicted to be low risk by either model had low rates of recurrence (<3%), and the deep learning pathology model identified a sizeable proportion of patients classified as high risk by the quantitative clinical models who could be reclassified as low risk (37% of high risk patients for the ER/PR model and 32% for the ER/PR/Ki-67 model). In an exploratory analysis, we evaluate how quantitative immunohistochemistry and Oncotype could be used together to improve prediction of recurrence. We first applied the 90% sensitivity threshold from above to further stratify risk after ODX testing (Supplementary Fig. 7). No patients with high ODX scores who were low-risk per the quantitative ER/PR or ER/PR/Ki-67 models experienced disease recurrence, and patients with low ODX but high-risk per the quantitative ER/PR/Ki-67 model had a trend towards worse recurrence-free interval (aHR 5.89, 95% CI 0.65–53.73, *p* = 0.12). We also assessed a model trained to directly predict recurrence in the University of Chicago cohort using Oncotype and features from the quantitative ER/PR/Ki-67 model (Supplementary Table 8). Incorporation of Oncotype with these features improved prediction of recurrence (c-index 0.85) over the quantitative ER/PR/Ki-67 model (c-index 0.71) or Oncotype alone (c-index 0.68), suggesting that more accurate prognostic models can be created through combination of Oncotype with quantitative IHC—although this combined model was fit and evaluated in the same cohort, so further validation is needed.

**Table 3:** Prognostic value of models in the University of Chicago Medical Center Validation Cohort

		RFI			RFS			OS		
	Model	aHR (CI)	*p*	c-index	aHR (CI)	*p*	c-index	aHR (CI)	*p*	c-index
**90% Sensitivity Threshold**	**No quantitative data**	2.86 (1.06–7.68)	0.04	0.65	1.27 (0.76–2.11)	0.36	0.63	1.19 (0.66–2.16)	0.56	0.75
	**Quant ER/PR**	2.96 (1.02–8.58)	0.05	0.65	1.12 (0.68–1.83)	0.66	0.63	0.98 (0.55–1.76)	0.95	0.75
	**Quant ER/PR/Ki-67**	3.84 (1.48–9.97)	0.01	0.68	1.13 (0.70–1.81)	0.63	0.63	0.84 (0.48–1.48)	0.55	0.75
**95% Sensitivity Threshold**	**No quantitative data**	1.41 (0.49–4.11)	0.53	0.59	0.8 (0.45–1.42)	0.45	0.64	0.83 (0.42–1.65)	0.60	0.76
	**Quant ER/PR**	3.39 (0.79–14.53)	0.10	0.62	1.08 (0.61–1.94)	0.79	0.63	0.92 (0.48–1.77)	0.8	0.75
	**Quant ER/PR/Ki-67**	3.64 (1.08–12.24)	0.04	0.65	1.11 (0.67–1.86)	0.69	0.63	0.92 (0.51–1.66)	0.78	0.75
**Raw Model Prediction**	**No quantitative data**	1.33 (1.03–1.73)	0.03	0.66	1.10 (0.86–1.42)	0.44	0.64	1.1 (0.79–1.54)	0.56	0.75
	**Quant ER/PR**	1.44 (1.09–1.89)	0.01	0.69	1.18 (0.92–1.51)	0.20	0.65	1.1 (0.78–1.54)	0.59	0.75
	**Quant ER/PR/Ki-67**	1.43 (1.11–1.85)	0.01	0.71	1.24 (0.98–1.57)	0.07	0.66	1.18 (0.84–1.65)	0.34	0.76

Associations of model predictions with recurrence-free interval (RFI), recurrence-free survival (RFS), and overall survival (OS) are shown for patients in the University of Chicago Medical Center cohort (*n* = 964). Results are listed for raw model predictions (normalized by standard deviation), as well as discretized predictions for high versus low risk (using thresholds achieving 90% or 95% sensitivity for high Oncotype DX score in the training cohort). In each case, the patient’s Charlson-Deyo comorbidity index and the actuarial life expectancy were included as covariates in a Cox proportional hazards model, with the adjusted hazard ratio (aHR) for model predictions, associated *p*-value, and concordance index (c-index) shown below.

## Discussion

Considering the high cost of ODX testing, machine learning methods have emerged as a potential tool for cost-effective prediction of a patient’s recurrence risk using routinely available clinical features. Here we demonstrate that, when predicting high-risk ODX status in patients with breast cancer, logistic regression models trained on a large NCDB dataset incorporating quantitative features for ER/PR% and Ki-67% outperform models utilizing only categorical features for ER/PR status. Our models achieved strong AUROCs at a level of performance comparable or better than models presented in other studies^18–20,22,25^. These performance improvements were preserved when validating our models on a diverse cohort of patients at UCMC.

Correlations between standard histopathologic variables and ODX score were first demonstrated in 2008 by Flanagan et. al in the Magee equations, which were later updated by Klein et al. in 2013^15,26^. Though the original equation utilized grade, HER2 status, ER/PR expression using the semi-quantitative IHC score (H-score), later versions included Ki-67 index. However, H-scores, which range from 0 to 300, are not universally reported, limiting their use. Quantitative components of grade are unavailable in NCDB, limiting direct comparison to the Magee equations.

Orucevic et al. previously developed the University of Tennessee nomogram for ODX using NCDB data incorporating tumor size, grade, PR status, and histologic subtype^22^. However, quantitative values for ER/PR expression were not available at the time of development of this nomogram. Yoo et al. and Kim et al. also developed logistic regression models incorporating nuclear grade, PR status, and Ki-67%^19,25^. In another study, Kim et al. applied decision jungles and neural networks for the prediction of high-risk ODX using ER/PR status, HER2 status, Ki-67 index, grade, and histologic subtype^20^. Moreover, when applying the University of Tennessee nomogram to a cohort of South Korean patients, Kim et al. demonstrated marked reductions in model performance^27^. These discrepancies may be suggestive of poor generalizability to Asian populations. Our model had equivalent performance in different racial/ethnic groups and maintained strong performance in a diverse validation cohort, which could contribute to the pursuit of health equity in clinical decision making.

Finally, our model was not only predictive of ODX score, but also long-term recurrence, which has rarely been evaluated in other studies^28^. Both quantitative models identified subsets of patients with very low risk of recurrence, with more low-risk patients identified when incorporating Ki-67. Nonetheless, our validated quantitative ER/PR model maintained higher accuracy for ODX than our non-quantitative model, and can identify a substantial proportion of patients as low-risk in settings where Ki-67 is not routinely obtained.

Despite strong evidence supporting the clinical utility of the ODX test, its high cost can be prohibitive in low-resource settings in the US as well as in other countries, and several studies have shown disparities in testing uptake associated with patient socioeconomic status^29–31^. Given the associated morbidity of adjuvant chemotherapy, there is particular interest in de-escalation of treatment in low-risk breast cancer patients for whom chemotherapy may not only fail to provide additional benefit but also introduce added toxicity and financial burden. The annual cost of ODX testing in the US is projected to increase to $231 million, and the use of highly sensitive cutoffs with computational models based on readily available clinicopathologic features could potentially reduce rates of ODX testing among patients unlikely to have positive results^32^. Furthermore, a number of recent studies have shown varying ability to predict ODX from digital histology^24,33–36^. We demonstrated here that our previously published deep learning model has similar performance to the quantitative clinical models detailed in this study and may have additive value to quantitative immunohistochemistry, as has been previously suggested^34^. Additionally, imaging-based radiomics approaches have also shown promise in augmenting predictions of a patient’s breast cancer recurrence risk, and may be incorporated in conjunction with clinical / pathologic features in future studies^37–41^. Finally, although models such as RSClin have been developed that may improve upon the prognostic accuracy of OncotypeDX by including additional clinical variables^42,43^, no such model incorporates standard quantitative immunohistochemistry. In our exploratory analyses we demonstrate that quantitative immunohistochemistry may have additive value to OncotypeDX testing, and larger well-annotated datasets are needed to confirm this finding and produce the next generation of risk-stratification tools.

There are several limitations to this study. The NCDB cohort captures the data of only patients for whom ODX testing was ordered, which may lead to bias related to practice patterns—such as underrepresentation of older patients who are not chemotherapy candidates. Quantitative values for percentage ER/PR expression have only been available through the NCDB in the past few years, limiting our ability to identify long-term differences in survival between low- and high-risk groups. Potential errors due to miscoding, lack of follow-up, and variability between sites may impact the quality of the NCDB data used to train the model. Furthermore, the training dataset was highly imbalanced with only 7% of cases classified as high-risk per OncotypeDX, (which is similar to other studies—for example, in RxPONDER trial, 10% of patients were excluded for high-risk OncotypeDX scores—and thus represents the national rate of high-risk disease^3,6^). Although under- or over-sampling is sometimes recommended for skewed data^44^, such approaches would impair our ability to accurately estimate risk as applicable in national cohorts, and despite skewed training data our models demonstrated strong calibration with true OncotypeDX scores across a spectrum of risk. Despite these limitations, our results were preserved in a single institution cohort with longer term follow-up, suggesting strong external validity and robustness.

Additionally, this study was tuned to predict a risk-of-recurrence assay in a nationally representative cohort from the United States, and although it is appealing to use such a tool in countries where genomic testing is unavailable, validation must be performed to ensure predictions remain robust due to differing demographics. Reassuringly, model accuracy remained robust across racial / ethnic groups despite the majority of training data coming from non-Hispanic white patients, which may indicate generalizability to populations worldwide. While less costly than genomic testing, even the quantitative immunohistochemistry necessary for these models is not uniformly available worldwide—surveys of facilities in sub-Saharan African report only 74–95% of centers utilize immunohistochemistry^45,46^, and Ki-67 may only be available in half of these centers. Nonetheless, the increase in automated tools for quantification of immunohistochemistry may facilitate the use of our risk prediction models^47,48^.

In conclusion, using training data from a large dataset of NCDB patients and validation data from a diverse cohort of UCMC patients, we have developed a machine learning model for the prediction of high-risk ODX score from clinicopathologic features which was prognostic for recurrence in an external dataset. This model may assist in the identification of low-risk patients who may safely refrain from adjuvant chemotherapy without further genomic testing.

## Methods

### Cohort selection

Data were extracted from the NCDB for new cases of invasive, HR-positive, HER2-negative, Stage I-III breast cancer in patients with diagnoses made between 2018 and 2020. Patients were also excluded if they had greater than three lymph nodes positive (reflective of current indications for ODX testing per guidelines) or missing ODX scores (Supplementary Fig. 8). An external validation cohort was identified from UCMC, including 970 invasive, HR-positive, HER2-negative, Stage I-III breast cancer patients diagnosed between 2006 through 2023, of which 305 patients had ODX scores. Association of baseline demographic variables with ODX result was assessed using a two-sided *t*-test for numeric variables and a chi-squared test for categorical variables.

### Model training

NCDB data were used to train and evaluate the performance of a machine learning model that predicted high- or low-risk ODX scores, with high-risk defined as scores of 26 or greater^49^. A subset comprising 80% of patients was used for model training, while the remaining data were set aside for internal validation. An overview of methods, model training, and model assessment is presented in Supplementary Fig. 9. After inspection of the log-likelihood ratios (LLRs) of the features in the original dataset, features with the greatest LLR were selected as a minimal potential feature set, while the remaining features were excluded (Supplementary Fig. 1). Any patients with missing data in the minimal feature set were excluded from further analysis. We evaluated the performance of four machine learning models—logistic regression, multilayer perceptron, random forest, and AdaBoost to predict high vs low ODX score using 10-fold cross-validation across a limited set of hyperparameters^50,51^. An informative set of features was selected for each model with sequential forward feature selection, using Akaike information criterion (AIC) as the metric (Supplementary Fig. 2)^52^. No model out-performed logistic regression with an increase of AUROC of >0.01 on cross validation in the training dataset, so a logistic regression base was used for further analysis given the greater interpretability of logistic regression models (Supplementary Table 9). To assess the comparative value of quantitative clinical models and models trained on digital histology, we also evaluated our previously published model to predict ODX score trained on breast cancer samples from The Cancer Genome Atlas^24^.

### Model assessment

We compared model performance between models with quantitative immunohistochemistry to a model without quantitative features. In the held-out test set from NCDB and the external validation cohort, confidence intervals and *p*-values for statistical significance of AUROC differences were computed using DeLong’s method; AUPRC confidence intervals were computed using bootstrapping with 1000 iterations^53,54^. Cutoffs with 95% and 90% sensitivity for high-risk ODX scores were computed in the NCDB training cohort, and model performance metrics were computed at each cutoff, including sensitivity, specificity, negative predictive value (NPV), and positive predictive value (PPV). Performance of the model was further evaluated by examining the correlation between model prediction and the patient’s known ODX score, and calibration plots were also generated based on the predicted and true probability of a high-risk ODX score in the NCDB test set. All statistical testing was performed at the 0.05 significance level, and all analysis was performed in Python 3.10.6 using sci-kit learn 1.2.1, lifelines 0.27.0, and SciPy 1.9.3.

### Survival analysis

Long-term survival outcomes were examined with Kaplan–Meier analysis, and hazard ratios and the Harrell’s concordance index (c-index) were calculated and compared between quantitative and non-quantitative models using Cox proportional hazards regression^55–57^. Charlson-Deyo comorbidity index and actuarial life expectancy were included as covariates for the Cox models^58^. Associations of model predictions with overall survival were analyzed in the NCDB cohort, whereas associations with overall survival, recurrence-free interval, and recurrence-free survival were analyzed in the UCMC cohort. Kaplan–Meier curves were generated using both 95% and 90% sensitivity thresholds to evaluate the model’s utility as a rule-out test, and the survival analysis was also performed using raw model predictions (normalized by standard deviation).

### Reporting summary

Further information on research design is available in the Nature Research Reporting Summary linked to this article.

### Supplementary information

Supplemental Materials

Reporting Summary

## Data Availability

The NCDB dataset is available to investigators from Commission on Cancer accredited cancer programs who complete an application process https://ncdbapp.facs.org/puf/. Data from the validation cohort is available from the authors upon reasonable request.

## Code availability

The code for data preprocessing, model training, and model assessment is provided as a Jupyter notebook. It was written in Python 3.10.6 and is publicly available as a repository on GitHub at github.com/fmhoward/NCDBRS.
