# Supplementary material for: Development and validation of a clinical breast cancer tool for accurate prediction of recurrence
Source: NPJ Breast Cancer. 2024 Jun 15;10:46. doi: 10.1038/s41523-024-00651-5 (PMC11180107; doi:10.1038/s41523-024-00651-5)
Supplement: Supplementary file 1 — Supplemental Materials [file 41523_2024_651_MOESM1_ESM.pdf]

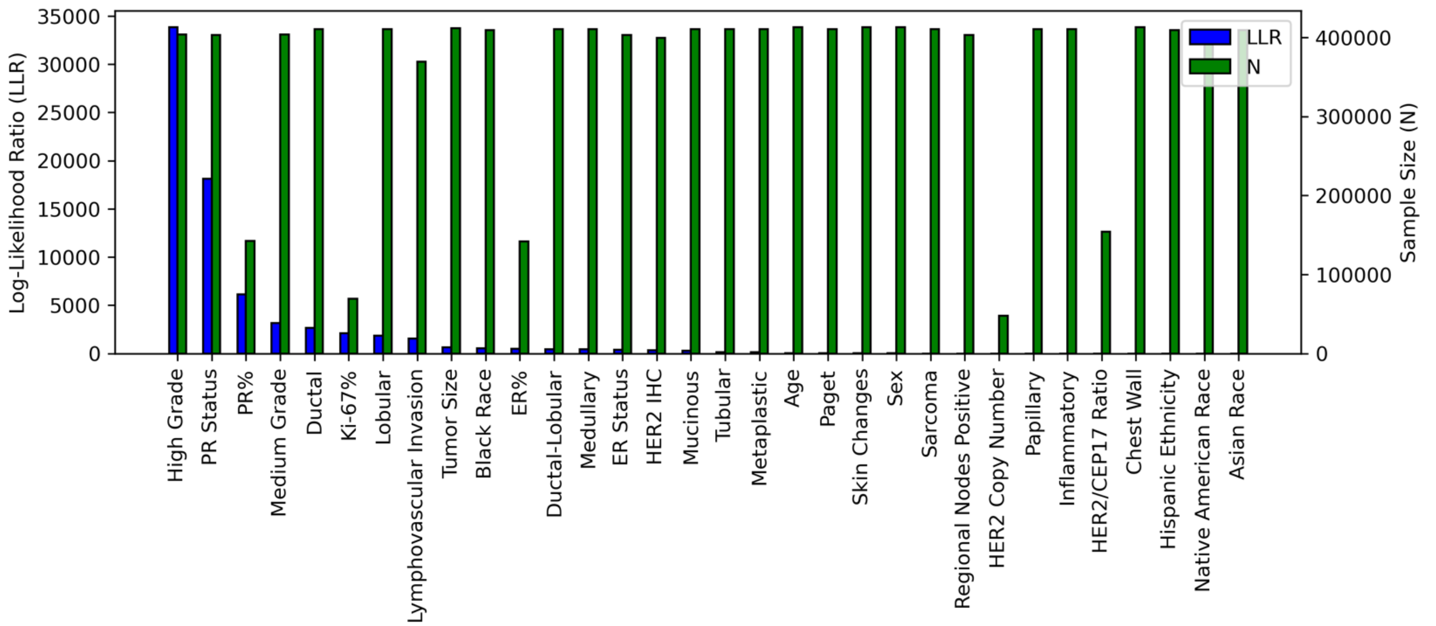

Supplementary Figure 1: Candidate Feature Selection. Results are shown for the log-likelihood ratios (LLR) and sample size (N) of patients with available data for the features in the National Cancer Database. Features are listed in descending order according to LLR. OF note, HER2 copy number and HER2/CEP17 ratio were excluded from the final feature set given the low predictive value and relative sparsity of data.

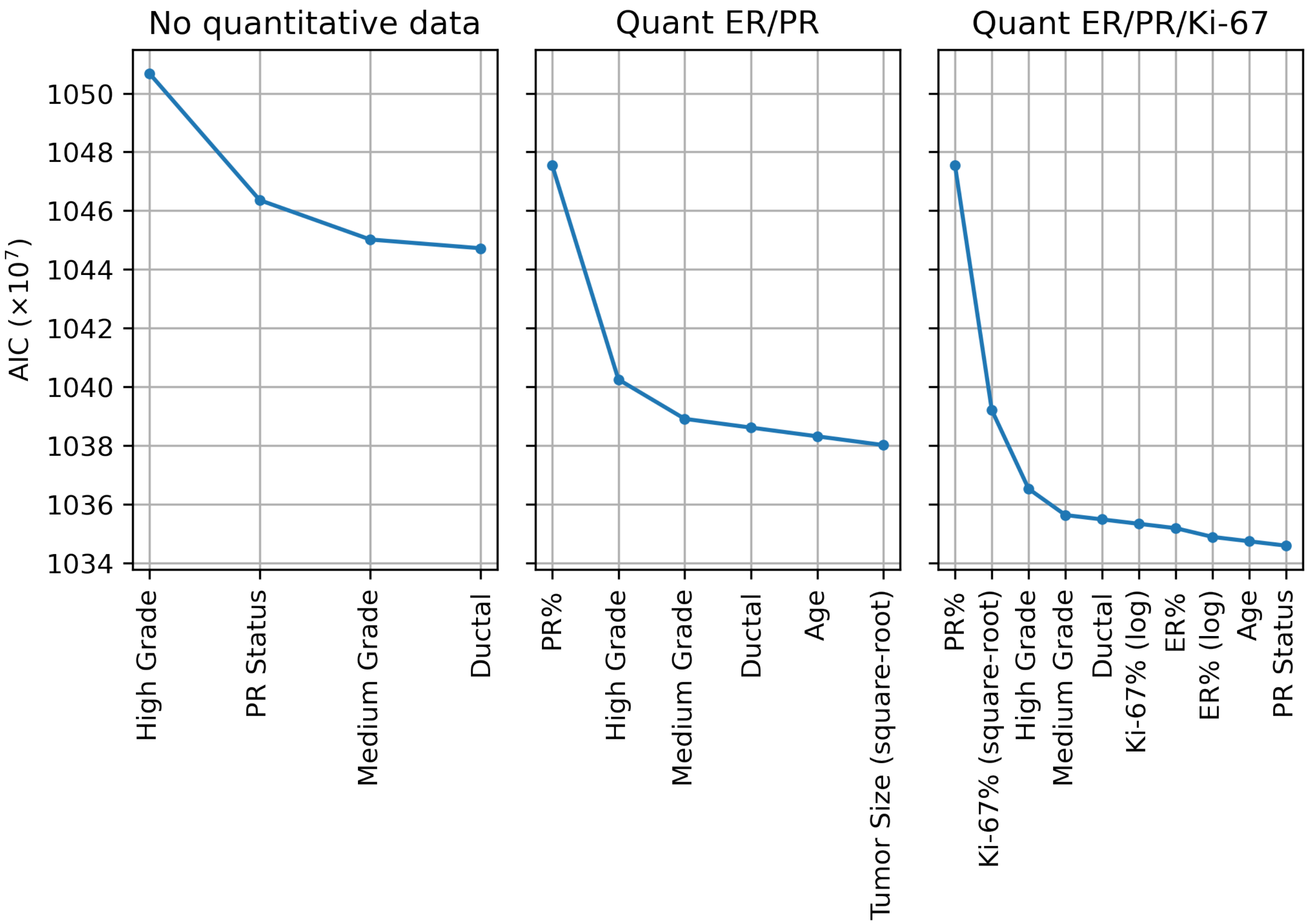

Supplementary Figure 2: Feature Selection. Models were trained using sequential forward feature selection to minimize the Akaike Information Criterion (AIC). For each model, results are shown for the most informative feature identified in each iteration of model training.

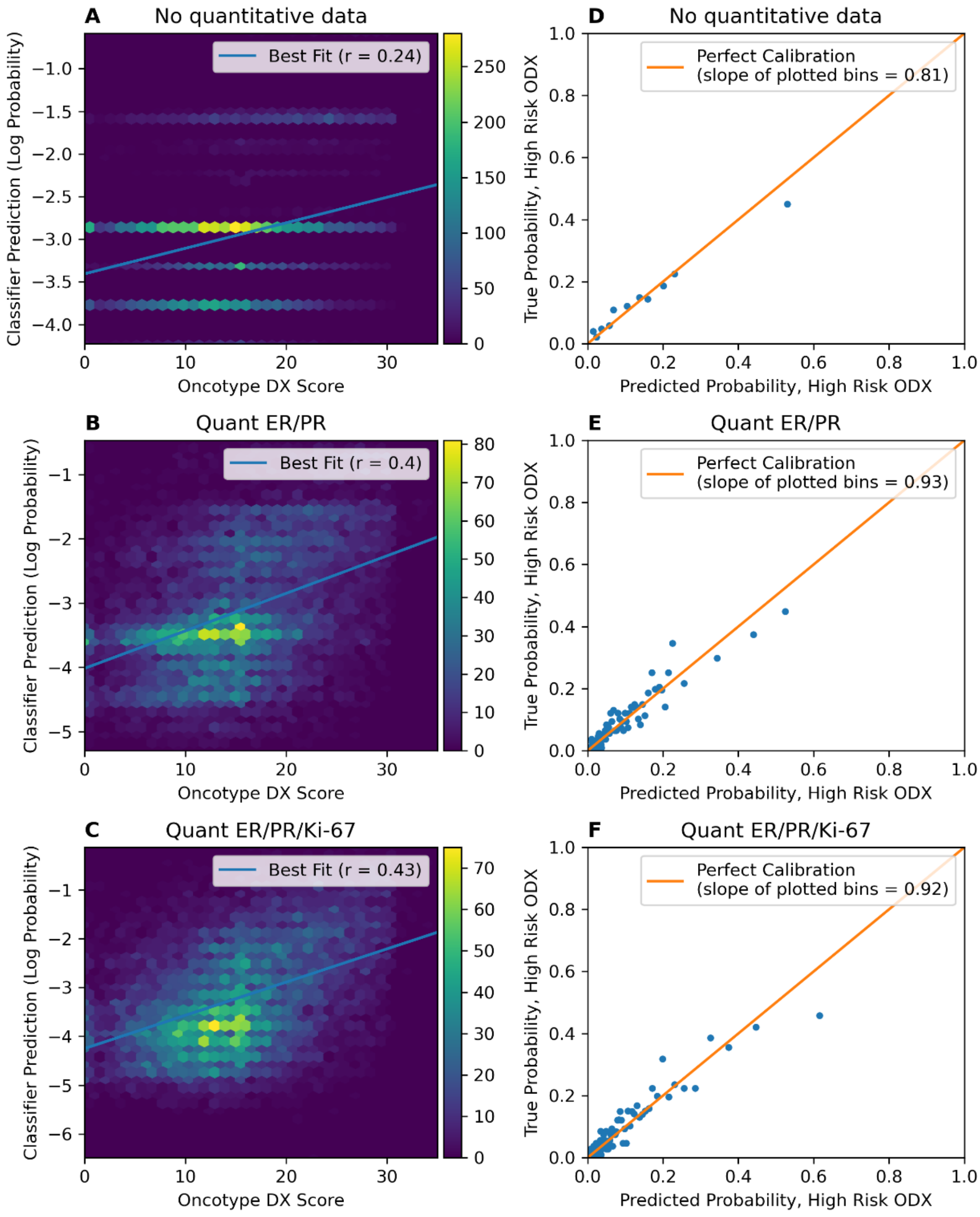

Supplementary Figure 3: Correlation and Calibration of Model Predictions. Density plots are shown for the association between true Oncotype DX score and the log-probability of classifier predictions in the held out NCDB test cohort for the **A.** non-quantitative model, **B.** quantitative ER/PR model, and **C.** quantitative ER/PR/Ki-67 model. In each case, the line of best fit is plotted along with the Pearson correlation coefficient. Calibration curves are also generated for the predicted and true probability of a high-risk Oncotype DX score for these same models, shown in **D**, **E**, and **F**, respectively. In each case, the line of best fit is plotted along with the slope.

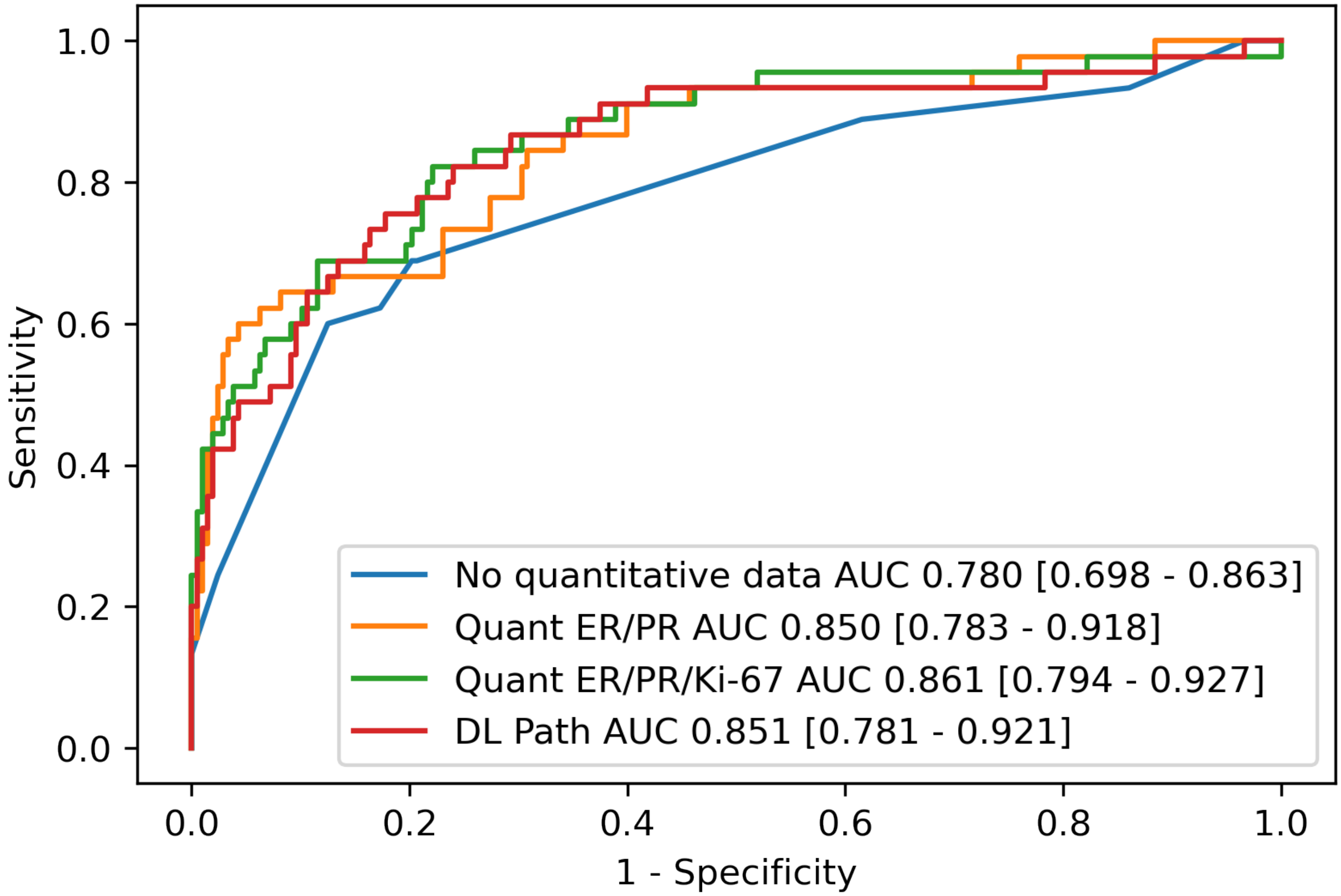

Supplementary Figure 4: Comparison of Quantitative Models to a Deep Learning Model Utilizing Digital Histology. Performance is shown in the subset of 253 patients from the University of Chicago validation cohort with quantitative immunohistochemistry as well as digital histology available.

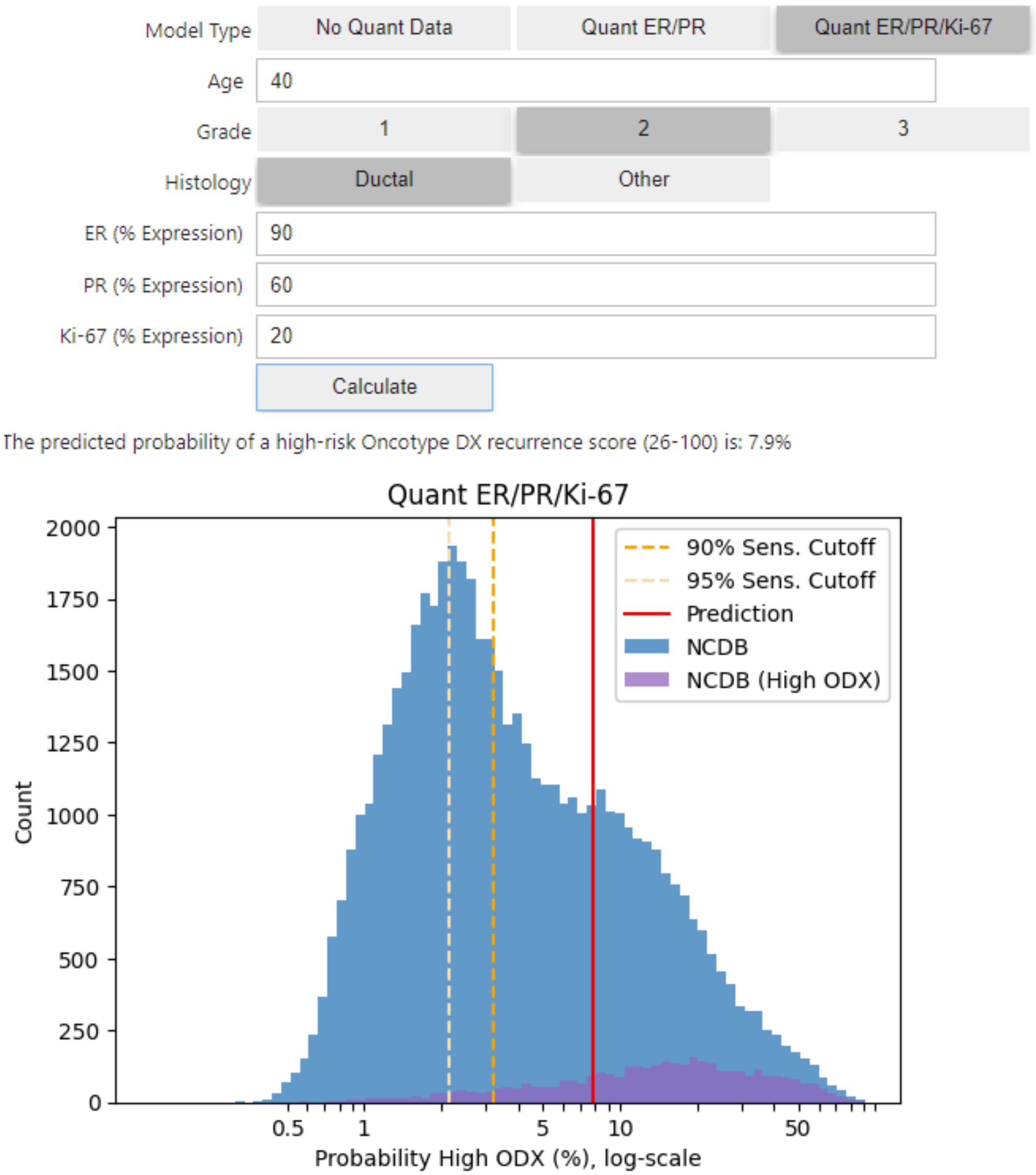

Supplementary Figure 5: Online Calculator to Facilitate Further Study of Model Predictions. Shown is example output of the online calculator for a 40-year-old patient with a grade 2 ductal carcinoma with ER 90%, PR 60%, and Ki-67 20% expression. Predictions can be displayed for the quantitative ER/PR model as well as the model without quantitative immunohistochemistry, to facilitate study in a variety of practice settings.

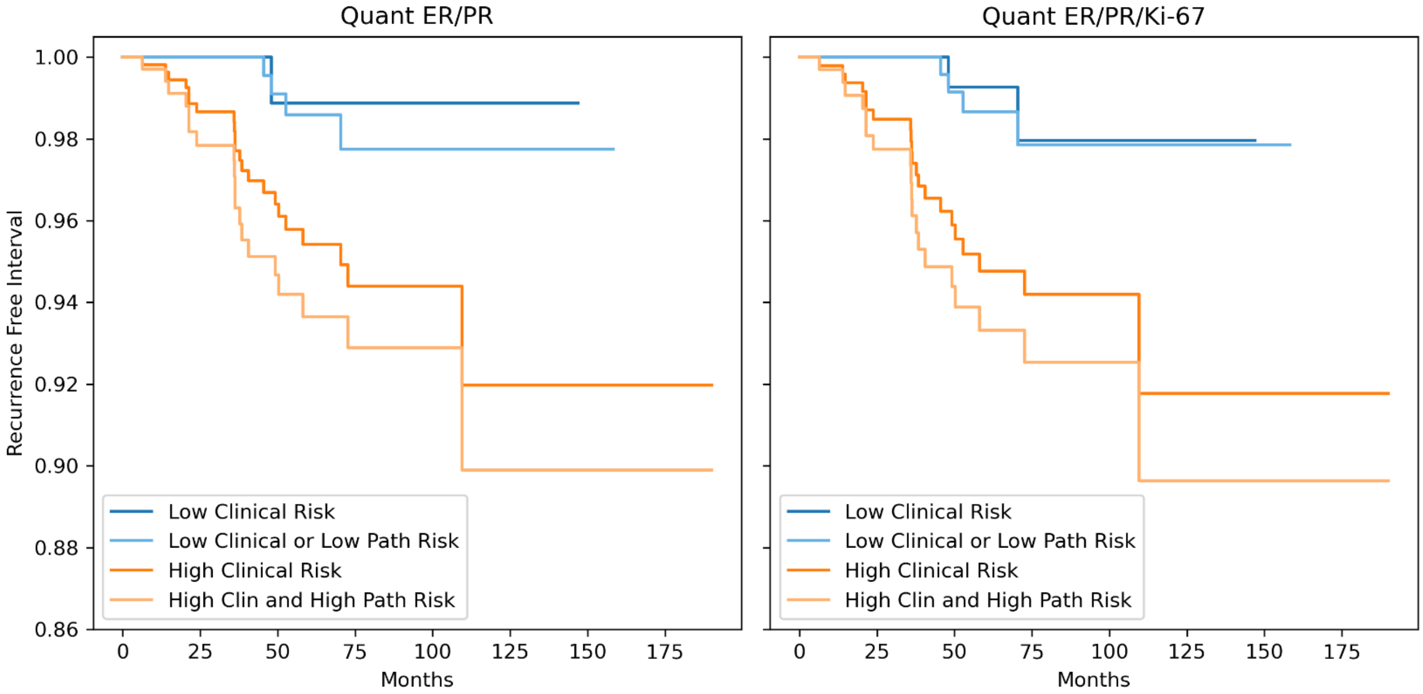

Supplementary Figure 6: Recurrence-Free Interval in Patients based on Risk Predicted by both Quantitative Clinical Models and a Deep Learning Model Utilizing Digital Histology. Performance is shown in the subset of patients (n = 670) from the University of Chicago Medical Center validation cohort with quantitative immunohistochemistry as well as digital histology available. Survival outcomes are similar in patients defined as low risk by the clinical model alone, or low risk by both models, but utilizing both models increases the percentage identified as low risk from 19% to 49% for the ER/PR model, and from 28% to 51% for the ER/PR/Ki-67 model.

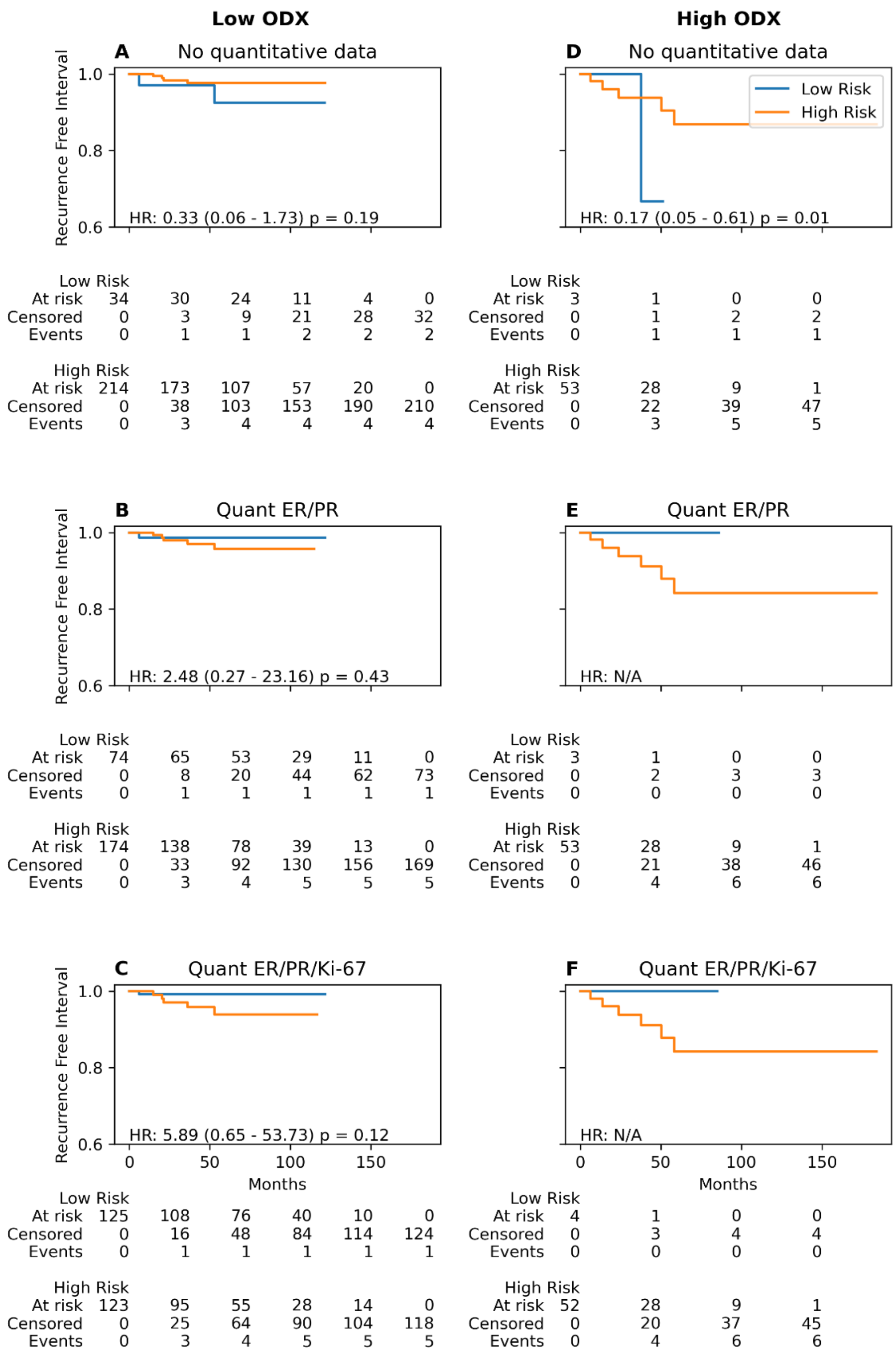

Supplementary Figure 7: Recurrence in Low- and High-Oncotype DX (ODX) Stratified by Model Predictions in the University of Chicago Medical Center Cohort. In patients from the University of Chicago validation cohort with low ODX (n = 249), Kaplan-Meier curves for recurrence-free interval are shown for patients classified as low- and high-risk by the **A.** non-quantitative model, **B.** quantitative ER/PR model, and **C.** quantitative ER/PR/Ki-67 model at the 90% sensitivity cutoff. Survival analysis results in patients with high ODX (n = 56) for these same models, shown in **D**, **E**, and **F**, respectively. Of note, no patients with high ODX identified as low risk by the quantitative models experienced disease recurrence.

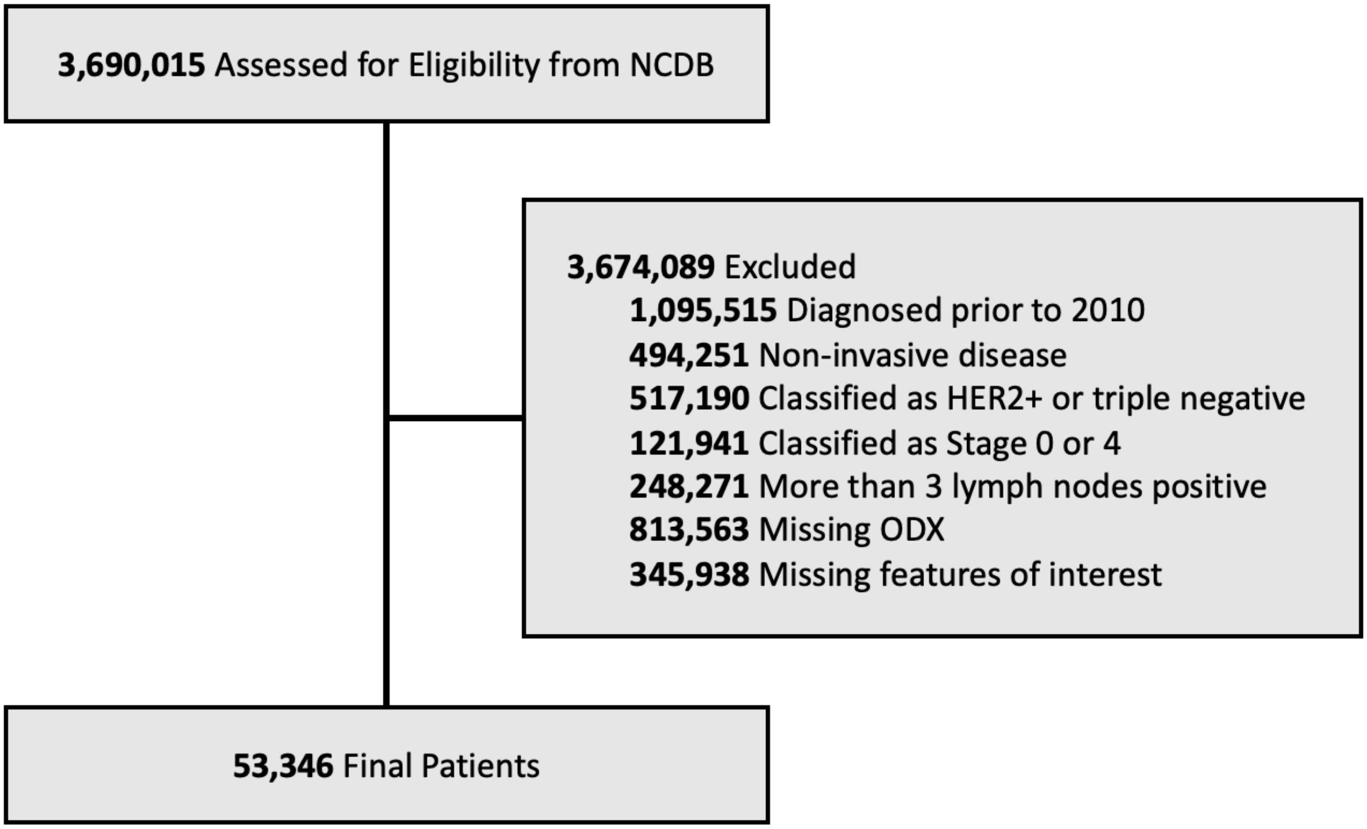

Supplementary Figure 8: National Cancer Database (NCDB) Cohort Selection Criteria

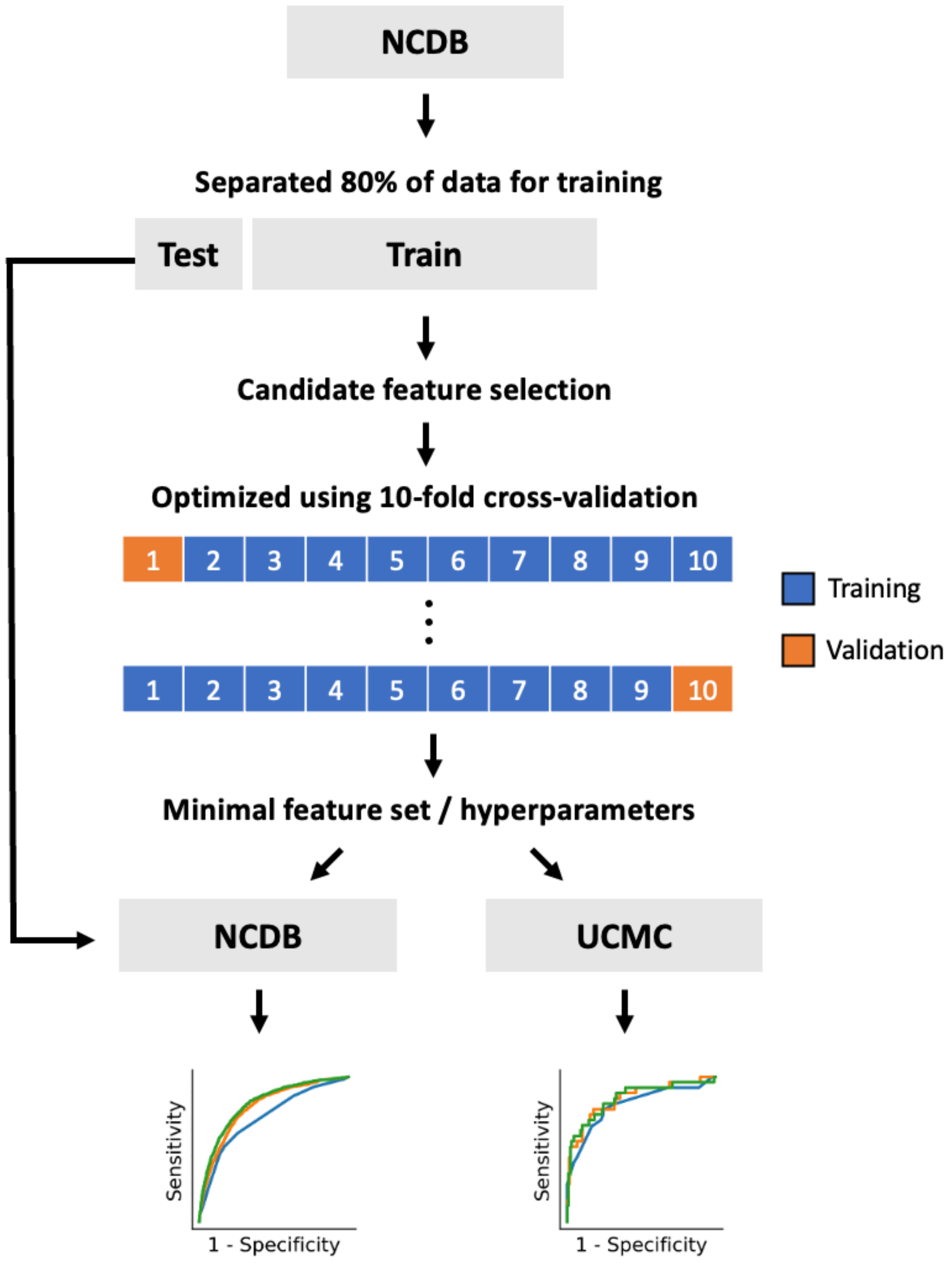

Supplementary Figure 9: Overview of Methods. Using the National Cancer Database (NCDB), a set of the most informative candidate features was determined to identify a cohort of patients (n = 53,346) without any missing data. A subset comprising 80% of patients was used for model training, while the remaining data were set aside for internal validation. This cohort was used to train logistic regression models using 10-fold cross-validation. Once trained, these models were applied back to the NCDB cohort for internal assessment of model performance. Additionally, these models were applied to an external cohort of patients from University of Chicago Medical Center (UCMC, n = 964 for survival outcomes, 305 with Oncotype DX available) for further validation.

Supplementary Table 1: Baseline Demographics from University of Chicago Medical Center Cohort used for Model Validation.

		Missing	Overall
**n**			970
**Age, mean (SD)**		0	58.4 (11.9)
**Sex, n (%)**	Female	0	970 (100.0)
**Race/Ethnicity, n (%)**	Asian	2	43 (4.4)
	Hispanic		33 (3.4)
	Non-Hispanic Black		298 (30.8)
	Non-Hispanic White		594 (61.4)
**Charlson-Deyo Score, n (%)**	0	0	800 (82.5)
	>= 1		170 (17.5)
**Grade, n (%)**	1	0	183 (18.9)
	2		555 (57.2)
	3		232 (23.9)
**Histologic Subtype, n (%)**	Ductal	11	723 (75.4)
	Ductal and Lobular		83 (8.7)
	Lobular		139 (14.5)
	Mucinous		7 (0.7)
	Others		7 (0.7)
**Tumor Size (mm), mean (SD)**		0	21.1 (18.5)
**Lymph Node Status, n (%)**	Negative	0	706 (72.8)
	Positive		264 (27.2)
**Lymphovascular Invasion, n (%)**	Absent	74	742 (82.8)
	Present		154 (17.2)
**Stage Group, n (%)**	1	0	637 (65.7)
	2		300 (30.9)
	3		33 (3.4)
**Receptor Status, n (%)**	ER+/PR+	0	835 (86.1)
	ER+/PR-		126 (13.0)
	ER-/PR+		9 (0.9)
**ER (% Positive), mean (SD)**		0	85.2 (20.7)
**PR (% Positive), mean (SD)**		0	58.5 (36.8)
**Ki-67 (% Positive), mean (SD)**		0	15.0 (17.2)
**Chemotherapy, n (%)**	Chemotherapy	0	285 (29.4)
	No Chemo		685 (70.6)
**Hormonal Therapy, n (%)**	Hormonal Therapy	0	905 (93.3)
	No Hormonal Therapy		65 (6.7)
**Vital Status, n (%)**	Alive	0	918 (94.6)
	Deceased		52 (5.4)
**Recurrence, n (%)**	No	6	935 (97.0)
	Yes		29 (3.0)
**Oncotype DX Score, n (%)**	High (26-100)	665	56 (18.4)
	Low-Intermediate (0-25)		249 (81.6)

Supplementary Table 2: Predictive Accuracy with Missing Model Inputs. Performance was recalculated presuming the listed model input features were missing, using mean imputation (with mean computed on training set) to generate a prediction without the listed variable. Results are listed for the area under the receiver operating characteristic curve (AUROC) and area under the precision recall curve (AUPRC) for prediction of high-risk Oncotype DX recurrence score. Confidence intervals and p-values for comparison to no missing features were generated using Delong's method for AUROC, and confidence intervals for AUPRC were generated using 1000 iteration bootstrapping.

		**National Cancer Database Held-Out Test Cohort**			**University of Chicago Validation Cohort**		
**Model**	**Missing Features**	**AUROC (CI)**	**AUPRC (CI)**	**P-value (vs None)**	**AUROC (CI)**	**AUPRC (CI)**	**P-value (vs None)**
**No quant data**	None	0.70 (0.68 - 0.72)	0.21 (0.18 - 0.23)	-	0.80 (0.73 - 0.87)	0.60 (0.47 - 0.71)	-
	PR Status	0.66 (0.64 - 0.68)	0.27 (0.25 - 0.29)	< 0.001	0.75 (0.68 - 0.83)	0.56 (0.45 - 0.67)	0.048
	Grade	0.58 (0.56 - 0.60)	0.20 (0.18 - 0.23)	< 0.001	0.69 (0.62 - 0.75)	0.48 (0.35 - 0.60)	0.001
	Histology	0.70 (0.68 - 0.72)	0.21 (0.18 - 0.23)	0.116	0.78 (0.70 - 0.85)	0.56 (0.44 - 0.67)	0.018
	Grade and PR Status	0.52 (0.50 - 0.53)	0.45 (0.43 - 0.46)	< 0.001	0.60 (0.54 - 0.65)	0.55 (0.50 - 0.60)	< 0.001
	Histology and PR Status	0.66 (0.64 - 0.67)	0.29 (0.26 - 0.31)	< 0.001	0.73 (0.66 - 0.80)	0.58 (0.47 - 0.67)	0.006
	Histology and Grade	0.57 (0.55 - 0.58)	0.23 (0.20 - 0.25)	< 0.001	0.62 (0.56 - 0.69)	0.47 (0.34 - 0.59)	< 0.001
**Quant ER/PR**	None	0.78 (0.77 - 0.80)	0.24 (0.22 - 0.27)	-	0.86 (0.80 - 0.92)	0.70 (0.56 - 0.80)	-
	PR %	0.66 (0.64 - 0.68)	0.14 (0.13 - 0.16)	< 0.001	0.75 (0.67 - 0.83)	0.42 (0.29 - 0.55)	0.001
	Grade	0.73 (0.71 - 0.75)	0.18 (0.16 - 0.20)	< 0.001	0.80 (0.74 - 0.87)	0.56 (0.42 - 0.68)	0.036
	Histology	0.78 (0.77 - 0.80)	0.24 (0.21 - 0.26)	0.974	0.84 (0.78 - 0.91)	0.64 (0.50 - 0.77)	0.049
	Age	0.78 (0.77 - 0.80)	0.24 (0.21 - 0.27)	0.808	0.86 (0.80 - 0.91)	0.67 (0.53 - 0.79)	0.680
	Tumor Size	0.78 (0.77 - 0.80)	0.24 (0.22 - 0.27)	0.064	0.86 (0.80 - 0.92)	0.71 (0.59 - 0.81)	0.015
	Grade and PR %	0.54 (0.52 - 0.56)	0.08 (0.07 - 0.09)	< 0.001	0.58 (0.50 - 0.66)	0.22 (0.15 - 0.30)	< 0.001
	Histology and PR %	0.66 (0.64 - 0.68)	0.14 (0.12 - 0.16)	< 0.001	0.72 (0.64 - 0.80)	0.40 (0.27 - 0.54)	< 0.001
	Age and PR %	0.67 (0.65 - 0.69)	0.15 (0.13 - 0.17)	< 0.001	0.75 (0.67 - 0.83)	0.41 (0.29 - 0.55)	0.001
	PR % and Tumor Size	0.66 (0.64 - 0.68)	0.14 (0.12 - 0.16)	< 0.001	0.76 (0.68 - 0.83)	0.44 (0.32 - 0.57)	0.002
	Histology and Grade	0.73 (0.71 - 0.75)	0.17 (0.15 - 0.19)	< 0.001	0.78 (0.70 - 0.85)	0.40 (0.28 - 0.51)	0.002
	Age and Grade	0.73 (0.72 - 0.75)	0.17 (0.16 - 0.19)	< 0.001	0.80 (0.74 - 0.87)	0.50 (0.37 - 0.62)	0.031
	Grade and Tumor Size	0.73 (0.71 - 0.75)	0.17 (0.15 - 0.19)	< 0.001	0.81 (0.75 - 0.88)	0.58 (0.44 - 0.71)	0.072
	Age and Histology	0.78 (0.77 - 0.80)	0.23 (0.21 - 0.26)	0.813	0.84 (0.78 - 0.90)	0.61 (0.46 - 0.75)	0.016
	Histology and Tumor Size	0.78 (0.77 - 0.80)	0.24 (0.21 - 0.27)	0.699	0.85 (0.79 - 0.92)	0.69 (0.56 - 0.81)	0.600
	Age and Tumor Size	0.78 (0.77 - 0.80)	0.25 (0.23 - 0.28)	0.291	0.86 (0.80 - 0.92)	0.69 (0.56 - 0.79)	0.645
**Quant ER/PR/ Ki-67**	None	0.81 (0.80 - 0.83)	0.28 (0.25 - 0.31)	-	0.87 (0.81 - 0.93)	0.71 (0.58 - 0.81)	-
	PR %	0.74 (0.72 - 0.75)	0.20 (0.18 - 0.23)	< 0.001	0.79 (0.72 - 0.87)	0.56 (0.42 - 0.69)	0.004
	Grade	0.79 (0.78 - 0.81)	0.25 (0.22 - 0.28)	< 0.001	0.85 (0.79 - 0.91)	0.63 (0.50 - 0.74)	0.109
	Histology	0.81 (0.80 - 0.83)	0.28 (0.25 - 0.31)	0.274	0.86 (0.80 - 0.92)	0.71 (0.59 - 0.81)	0.036
	ER %	0.81 (0.79 - 0.82)	0.28 (0.25 - 0.31)	0.002	0.87 (0.82 - 0.93)	0.70 (0.57 - 0.82)	0.316
	Age	0.81 (0.80 - 0.83)	0.28 (0.25 - 0.31)	0.978	0.87 (0.81 - 0.93)	0.70 (0.57 - 0.81)	0.278
	PR	0.72 (0.70 - 0.74)	0.19 (0.17 - 0.21)	< 0.001	0.78 (0.70 - 0.85)	0.53 (0.38 - 0.67)	0.002
	Ki67 %	0.79 (0.77 - 0.80)	0.25 (0.22 - 0.28)	< 0.001	0.85 (0.79 - 0.91)	0.68 (0.56 - 0.78)	0.124
	Grade and Ki67 %	0.73 (0.71 - 0.75)	0.18 (0.16 - 0.21)	< 0.001	0.79 (0.72 - 0.86)	0.51 (0.37 - 0.65)	0.003
	Histology and Ki67 %	0.79 (0.77 - 0.80)	0.24 (0.21 - 0.27)	< 0.001	0.84 (0.78 - 0.91)	0.66 (0.53 - 0.77)	0.023
	ER % and Ki67 %	0.78 (0.77 - 0.80)	0.24 (0.21 - 0.27)	< 0.001	0.86 (0.80 - 0.92)	0.69 (0.56 - 0.79)	0.352
	Age and Ki67 %	0.79 (0.77 - 0.80)	0.24 (0.22 - 0.28)	< 0.001	0.85 (0.79 - 0.91)	0.67 (0.55 - 0.77)	0.084
	Ki67 % and PR Status	0.68 (0.66 - 0.70)	0.16 (0.14 - 0.18)	< 0.001	0.76 (0.69 - 0.84)	0.50 (0.35 - 0.62)	0.001
	Ki67 % and PR %	0.70 (0.68 - 0.72)	0.18 (0.16 - 0.20)	< 0.001	0.79 (0.71 - 0.86)	0.56 (0.43 - 0.68)	0.004
	Grade and PR %	0.72 (0.70 - 0.74)	0.18 (0.16 - 0.20)	< 0.001	0.78 (0.71 - 0.85)	0.51 (0.38 - 0.64)	0.001
	Histology and PR %	0.74 (0.72 - 0.76)	0.20 (0.18 - 0.23)	< 0.001	0.79 (0.71 - 0.86)	0.57 (0.42 - 0.70)	0.001
	ER % and PR %	0.72 (0.70 - 0.74)	0.19 (0.17 - 0.21)	< 0.001	0.79 (0.72 - 0.87)	0.54 (0.40 - 0.67)	0.004
	Age and PR %	0.74 (0.72 - 0.76)	0.20 (0.18 - 0.23)	< 0.001	0.80 (0.72 - 0.87)	0.57 (0.43 - 0.70)	0.005
	Histology and Grade	0.80 (0.78 - 0.81)	0.25 (0.22 - 0.27)	< 0.001	0.84 (0.78 - 0.90)	0.60 (0.47 - 0.72)	0.022
	ER % and Grade	0.79 (0.77 - 0.81)	0.25 (0.22 - 0.28)	< 0.001	0.86 (0.81 - 0.92)	0.64 (0.52 - 0.75)	0.633
	Age and Grade	0.79 (0.78 - 0.81)	0.25 (0.22 - 0.28)	< 0.001	0.85 (0.79 - 0.91)	0.62 (0.49 - 0.74)	0.071
	Grade and PR Status	0.70 (0.68 - 0.72)	0.17 (0.15 - 0.19)	< 0.001	0.76 (0.68 - 0.83)	0.47 (0.33 - 0.60)	< 0.001
	Histology and ER %	0.81 (0.79 - 0.83)	0.28 (0.25 - 0.31)	0.212	0.86 (0.80 - 0.93)	0.70 (0.58 - 0.82)	0.328
	Age and Histology	0.81 (0.80 - 0.83)	0.28 (0.25 - 0.31)	0.390	0.86 (0.80 - 0.92)	0.70 (0.58 - 0.80)	0.020
	Histology and PR Status	0.73 (0.71 - 0.75)	0.19 (0.17 - 0.22)	< 0.001	0.77 (0.69 - 0.85)	0.54 (0.39 - 0.66)	< 0.001
	Age and ER %	0.81 (0.79 - 0.82)	0.28 (0.25 - 0.31)	0.004	0.87 (0.82 - 0.93)	0.70 (0.58 - 0.81)	0.531
	ER % and PR Status	0.71 (0.69 - 0.73)	0.18 (0.16 - 0.21)	< 0.001	0.77 (0.70 - 0.85)	0.52 (0.38 - 0.65)	0.002
	Age and PR Status	0.73 (0.71 - 0.74)	0.19 (0.17 - 0.22)	< 0.001	0.78 (0.70 - 0.86)	0.55 (0.40 - 0.69)	0.002

Supplementary Table 3: Predictive Accuracy in Histologic Subtypes. Results are listed for area under the receiver operating characteristic (AUROC) and area under the precision recall curve (AUPRC) for prediction of high-risk Oncotype DX recurrence score in the National Cancer Database held-out test dataset with 95% confidence intervals for ductal (n = 8,299), lobular (n = 1,657), ductal and lobular (n = 484), and mucinous (n = 187) histologic subtypes, demonstrating that performance is preserved in these subtypes. Other subgroups were not examined given small sample sizes limiting AUROC analysis.

		**AUROC (CI)**	**AUPRC (CI)**
**Model**	**Subgroup**		
**No quantitative data**	**Ductal**	0.71 (0.69 - 0.73)	0.23 (0.20 - 0.26)
	**Ductal and Lobular**	0.65 (0.58 - 0.73)	0.16 (0.09 - 0.26)
	**Lobular**	0.63 (0.57 - 0.69)	0.18 (0.11 - 0.26)
	**Mucinous**	0.46 (0.28 - 0.65)	0.07 (0.02 - 0.26)
**Quant ER/PR**	**Ductal**	0.80 (0.78 - 0.81)	0.26 (0.22 - 0.29)
	**Ductal and Lobular**	0.76 (0.68 - 0.83)	0.21 (0.13 - 0.32)
	**Lobular**	0.75 (0.69 - 0.80)	0.17 (0.12 - 0.23)
	**Mucinous**	0.66 (0.49 - 0.84)	0.11 (0.03 - 0.23)
**Quant ER/PR/Ki-67**	**Ductal**	0.82 (0.80 - 0.84)	0.30 (0.26 - 0.33)
	**Ductal and Lobular**	0.80 (0.73 - 0.87)	0.27 (0.15 - 0.39)
	**Lobular**	0.78 (0.73 - 0.83)	0.21 (0.15 - 0.28)
	**Mucinous**	0.74 (0.60 - 0.89)	0.12 (0.04 - 0.25)

Supplementary Table 4: Predictive Accuracy in Racial/Ethnic Subgroups. Results are listed for area under the receiver operating characteristic (AUROC) and area under the precision recall curve (AUPRC) for prediction of high-risk Oncotype DX recurrence score in the National Cancer Database held out test dataset with 95% confidence intervals for Asian (n = 499), Hispanic (n = 662), Non-Hispanic Black (n = 838), and Non-Hispanic White (n = 8,565) racial subgroups, demonstrating that performance is preserved in these subgroups. Other subgroups were not examined given small sample sizes limiting AUROC analysis.

		**AUROC (CI)**	**AUPRC (CI)**
**Model**	**Subgroup**		
**No quantitative data**	**Asian**	0.69 (0.60 - 0.77)	0.26 (0.14 - 0.41)
	**Hispanic**	0.71 (0.62 - 0.79)	0.23 (0.12 - 0.36)
	**Non-Hispanic Black**	0.70 (0.63 - 0.77)	0.24 (0.15 - 0.34)
	**Non-Hispanic White**	0.70 (0.68 - 0.72)	0.20 (0.17 - 0.23)
**Quant ER/PR**	**Asian**	0.78 (0.71 - 0.85)	0.23 (0.14 - 0.34)
	**Hispanic**	0.78 (0.70 - 0.86)	0.27 (0.15 - 0.41)
	**Non-Hispanic Black**	0.80 (0.75 - 0.84)	0.25 (0.16 - 0.34)
	**Non-Hispanic White**	0.78 (0.76 - 0.80)	0.24 (0.21 - 0.28)
**Quant ER/PR/Ki-67**	**Asian**	0.78 (0.71 - 0.86)	0.27 (0.16 - 0.39)
	**Hispanic**	0.81 (0.74 - 0.88)	0.31 (0.18 - 0.47)
	**Non-Hispanic Black**	0.83 (0.78 - 0.87)	0.27 (0.19 - 0.37)
	**Non-Hispanic White**	0.81 (0.79 - 0.83)	0.28 (0.25 - 0.32)

Supplementary Table 5: Predictive Accuracy in Nodal Subgroups. Results are listed for area under the receiver operating characteristic (AUROC) and area under the precision recall curve (AUPRC) for prediction of high-risk Oncotype DX recurrence score in the National Cancer Database held out test dataset with 95% confidence intervals for negative (n = 8,794) and positive (n = 1,876) nodal subgroups, demonstrating that performance is preserved in these subgroups.

		**AUROC (CI)**	**AUPRC (CI)**
**Model**	**Subgroup**		
**No quantitative data**	**Negative**	0.70 (0.68 - 0.72)	0.21 (0.18 - 0.24)
	**Positive**	0.71 (0.66 - 0.75)	0.19 (0.14 - 0.25)
**Quant ER/PR**	**Negative**	0.79 (0.77 - 0.81)	0.26 (0.23 - 0.29)
	**Positive**	0.76 (0.72 - 0.80)	0.20 (0.16 - 0.25)
**Quant ER/PR/Ki-67**	**Negative**	0.82 (0.80 - 0.83)	0.30 (0.26 - 0.34)
	**Positive**	0.79 (0.75 - 0.83)	0.23 (0.18 - 0.28)

Supplementary Table 6: Utility of Models as Rule-Out Tests. Using National Cancer Database (NCDB) training data (n = 42,676), thresholds were identified for 95% and 90% sensitivity for high-risk recurrence scores. These thresholds were applied to the NCDB test set (n = 10,670) and University of Chicago Medical Center (UCMC) cohort (n = 305) to evaluate performance characteristics, including sensitivity (Sen) specificity (Spe), positive predictive value (PPV), and negative predictive value (NPV).

		**NCDB Test Set**				**UCMC**			
	**Model**	**Sen**	**Spe**	**PPV**	**NPV**	**Sen**	**Spe**	**PPV**	**NPV**
**95% Sensitivity**	**No quantitative data**	0.91	0.27	0.09	0.98	0.95	0.14	0.20	0.92
	**Quant ER/PR**	0.94	0.29	0.09	0.99	0.98	0.23	0.22	0.98
	**Quant ER/PR/Ki-67**	0.95	0.36	0.10	0.99	0.96	0.34	0.25	0.98
**90% Sensitivity**	**No quantitative data**	0.82	0.41	0.10	0.97	0.91	0.38	0.25	0.95
	**Quant ER/PR**	0.88	0.47	0.12	0.98	0.95	0.33	0.24	0.96
	**Quant ER/PR/Ki-67**	0.90	0.52	0.13	0.99	0.93	0.54	0.31	0.97

Supplementary Table 7: Prognostic Value of Models in the National Cancer Database Cohort.

Associations of model predictions with recurrence-free interval (RFI), recurrence-free survival (RFS), and overall survival (OS) are shown for patients in the National Cancer Database test set (n = 10,670). Results are listed for raw model predictions (normalized by standard deviation), as well as discretized predictions for high versus low risk (using thresholds achieving 90% or 95% sensitivity for high Oncotype DX score in the training cohort). In each case, the patient's Charlson-Deyo comorbidity index and the actuarial life expectancy were included as covariates in a Cox proportional hazards model, with the adjusted hazard ratio (aHR) for model predictions, associated p-value, and concordance index (c-index) shown below.

		**aHR (CI)**	**c-index**	**p**
	**Model**			
**90% Sensitivity**	**No quantitative data**	1.13 (0.73 - 1.73)	0.71	0.59
	**Quant ER/PR**	1.48 (0.97 - 2.28)	0.71	0.07
	**Quant ER/PR/Ki-67**	1.36 (0.89 - 2.08)	0.71	0.15
**95% Sensitivity**	**No quantitative data**	1.14 (0.70 - 1.87)	0.71	0.59
	**Quant ER/PR**	1.22 (0.76 - 1.99)	0.71	0.41
	**Quant ER/PR/Ki-67**	1.11 (0.71 - 1.73)	0.71	0.64
**Raw Model Prediction**	**No quantitative data**	1.16 (0.99 - 1.35)	0.71	0.07
	**Quant ER/PR**	1.06 (0.87 - 1.28)	0.71	0.58
	**Quant ER/PR/Ki-67**	1.17 (0.99 - 1.39)	0.71	0.06

Supplementary Table 8: Direct Prediction of Recurrence Integrating Quantitative Features and Oncotype Score

Associations of quantitative ER/PR/Ki-67 model features for recurrence-free interval (RFI) are shown for models fit in the University of Chicago Medical Center cohort where recurrence information, Oncotype score, and covariates were known (n = 305). Results are first listed for univariable hazard ratios (HR) quantifying the association of each feature with RFI, as well as associated p-value for the parameter and concordance index, indicating that quantitative ER, quantitative Ki-67 and Oncotype score are each associated with recurrence. Additionally, a multivariable model was fit with all model features in combination with Oncotype, with adjusted hazard ratios (aHR) reported, demonstrating that quantitative Ki-67 was the only significant feature in the multivariable model. Of note, transformed versions of ER/PR/Ki-67 and qualitative PR status (as included in the fit ER/PR/Ki-67 model) were not included in this analysis due to small sample size for model fitting.

Feature	HR (CI)	c-index	p	aHR (CI)	c-index	p
**Age**	0.98 (0.92 - 1.03)	0.55	0.37	0.97 (0.91 - 1.04)	0.85	0.38
**Ductal Histology**	5.37 (0.69 - 41.63)	0.63	0.11	3.34 (0.41 - 26.97)	0.85	0.26
**High Grade**	2.11 (0.64 - 7.02)	0.58	0.22	0.11 (0.01 - 1.02)	0.85	0.05
**Intermediate Grade**	0.37 (0.12 - 1.16)	0.63	0.09	0.29 (0.07 - 1.30)	0.85	0.11
**ER %**	0.97 (0.95 - 0.99)	0.60	0.01	0.98 (0.96 - 1.00)	0.85	0.11
**PR %**	0.99 (0.97 - 1.00)	0.67	0.14	0.99 (0.98 - 1.01)	0.85	0.61
**Ki-67 %**	1.04 (1.01 - 1.06)	0.70	0.002	1.05 (1.01 - 1.08)	0.85	0.01
**OncotypeDX**	1.06 (1.03 - 1.10)	0.68	0.001	1.05 (0.98 - 1.12)	0.85	0.17

Supplementary Table 9: Grid Search and Hyperparameter Tuning. Select machine learning models, including logistic regression, random forest, AdaBoost, and neural networks, were explored as the model framework, with a grid search performed over a subset of hyperparameters to maximize area under the receiver operating characteristic curve (AUROC) of the quantitative ER/PR/Ki-67 model. For each model type, the hyperparameters and array of values chosen for the grid search are shown. The best set of hyperparameters and maximum AUROC achieved are shown in the final two columns.

Model	Hyperparameters	Values	Best Hyperparameters	Best AUROC
**Logistic Regression**	Penalty	None, L1, L2	L1	0.818
	C	0.01, 0.1, 1, 10	0.1	
	Fit Intercept	False, True	False	
**Random Forest**	Criterion	Gini, Entropy	Entropy	0.726
	Max Depth	None, 10, 50	10	
	Max Features	None, sqrt, log2	sqrt	
	Polynomial Degree	1, 2	2	
**AdaBoost**	Number of Estimators	1, 5, 10, 50, 100, 500, 1000	50	0.815
	Polynomial Degree	1, 2	1	
**Neural Network**	Hidden Layer Sizes	(10,), (50,), (100,), (10,10), (50,50), (100,100)	(50,)	0.819
	Activation	logistic, tanh, relu	logistic	
	Initial Learning Rate	0.01, 0.001, 0.0001	0.001	
	Alpha	0.0001, 0.00001	0.00001	
	Polynomial Degree	1, 2	1	
